# Role and Cytotoxicity of Amylin and Protection of Pancreatic Islet β-Cells from Amylin Cytotoxicity

**DOI:** 10.3390/cells7080095

**Published:** 2018-08-06

**Authors:** Yoshimitsu Kiriyama, Hiromi Nochi

**Affiliations:** Kagawa School of Pharmaceutical Sciences, Tokushima Bunri University, Shido 1314-1, Kagawa, Sanuki 769-2193, Japan; kiriyamay@kph.bunri-u.ac.jp

**Keywords:** amylin, IAPP, diabetes, autophagy, β-sheet breaker, chemical chaperone, foldamer

## Abstract

Amylin, (or islet amyloid polypeptide; IAPP), a 37-amino acid peptide hormone, is released in response to nutrients, including glucose, lipids or amino acids. Amylin is co-stored and co-secreted with insulin by pancreatic islet β-cells. Amylin inhibits food intake, delays gastric emptying, and decreases blood glucose levels, leading to the reduction of body weight. Therefore, amylin as well as insulin play important roles in controlling the level of blood glucose. However, human amylin aggregates and human amylin oligomers cause membrane disruption, endoplasmic reticulum (ER) stress and mitochondrial damage. Since cytotoxicity of human amylin oligomers to pancreatic islet β-cells can lead to diabetes, the protection of pancreatic islet β cells from cytotoxic amylin is crucial. Human amylin oligomers also inhibit autophagy, although autophagy can function to remove amylin aggregates and damaged organelles. Small molecules, including β-sheet breaker peptides, chemical chaperones, and foldamers, inhibit and disaggregate amyloid formed by human amylin, suggesting the possible use of these small molecules in the treatment of diabetes. In this review, we summarize recent findings regarding the role and cytotoxicity of amylin and the protection of pancreatic islet β-cells from cytotoxicity of amylin.

## 1. Introduction

**Figure 1:**
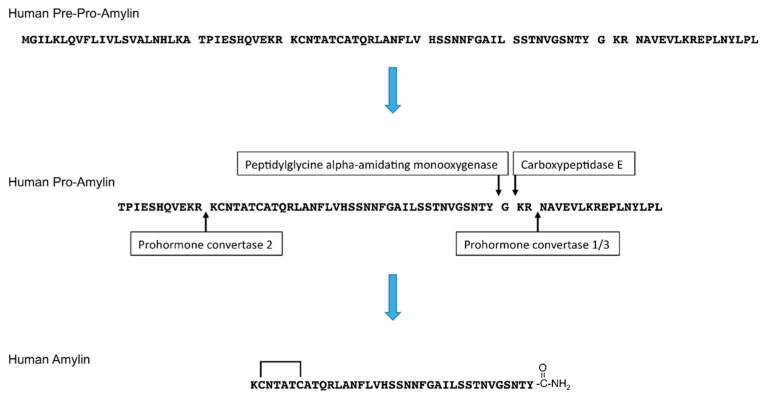
Amino acid sequences and processing of human pre-pro-amylin to generate mature human amylin. The N-terminal signal peptide is removed from the 89-amino acid pre-pro-amylin to produce the 67-amino acid pro-amylin. Pro-amylin is cleaved by prohormone convertase 1/3 and prohormone convertase 2, and the C-terminus of amylin is cleaved by carboxypeptidase E and amidated by peptidylglycine alpha-amidating monooxygenase. A disulfide bond at the N-terminus of amylin is subsequently formed.

Amylin, also known as islet amyloid polypeptide (IAPP), is a 37-amino acid peptide hormone. Amylin is released in response to nutrients, including glucose, lipids, or amino acid (arginine) [1,2]. Pancreatic islets include α, β, δ, γ/pancreatic polypeptide (PP), and ε cells. α cells secrete glucagon, β-cells secrete insulin and amylin, δ cells secrete somatostatin, γ/PP cells secrete pancreatic polypeptide, and ε cells secrete ghrelin [3,4]. Amylin is co-stored and co-secreted with insulin by pancreatic islet β-cells [5,6,7]. After removal of the signal peptide, the newly synthesized 89-amino acid pre-pro-hormone becomes pro-amylin, which is 67-amino acid prohormone. Then, pro-amylin is processed by prohormone convertase (PC) 1/3 and PC2, and its C-terminus is amidated by carboxypeptidase E and peptidylglycine alpha-amidating monooxygenase. A disulfide bond is formed at its N-terminus [8,9] (Figure 1).

**Figure 2:**
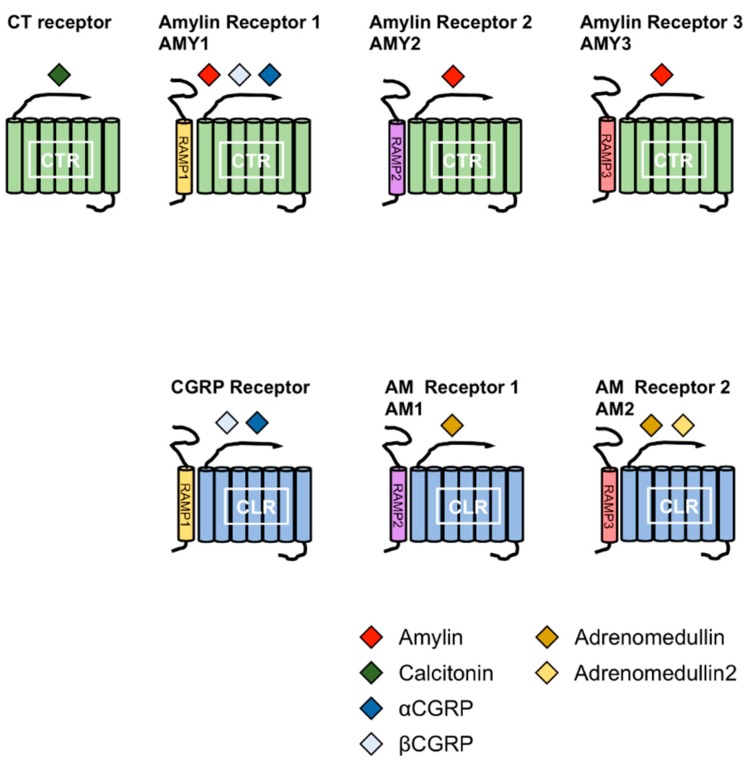
Combination of receptor activity-modifying proteins (RAMPs) and calcitonin receptor (CTR) or CLR leads to the formation of different receptors for calctionin, CGRPs, amylin and adrenomedullins. The CT family comprises amylin, calcitonin (CT), αcalcitonin gene-related peptide (αCGRP), βCGRP, adrenomedullin (AM) and AM2. The combination of RAMPs and CTR or CTR-like receptor (CLR) leads to the formation of different receptors for the members of the CT family. Amylin receptors include AMY_1_, AMY_2_ and AMY_3_ receptors comprising the CTR complex to RAMP1, RAMP2 and RAMP3, respectively.

Amylin is a member of the calcitonin (CT) family, which includes CT, αcalcitonin gene-related peptide (αCGRP), βCGRP, adrenomedullin (AM), and AM2 (also known as intermedin). The members of this family comprise a disulfide bond at the N-terminus and an amidated C-terminus. CT receptor (CTR) and CTR-like receptor (CLR) are receptors for the members of CT family. CTR has splice variants with tissue-specific expression patterns [10,11,12,13]. Receptor activity-modifying proteins (RAMPs) are associated with CTR or CLR. To date, three RAMPs have been reported, and the combination of CTR or CLR and RAMPs determines the specificity for the members of the CT family [12,14,15,16]. Amylin receptors are heterodimers of CTR and RAMPs. Currently, three receptors for amylin have been reported; AMY_1_, AMY_2_ and AMY_3_ receptors. AMY_1_ receptor consists of CTR and RAMP1, AMY_2_ receptor consists of CTR and RAMP2 and AMY_3_ receptor consists of CTR and RAMP3 (Figure 2).

Numerous proteins, including human amylin, aggregate and form amyloids. The formation of amyloids is associated with the induction of cytotoxicity, leading to diseases such as type 2 diabetes (T2D), Creutzfeldt–Jakob disease, Alzheimer’s disease, Parkinson’s disease and Huntington’s disease [17]. However, recent studies have shown that aggregates of certain proteins are not linked to cytotoxicity [18,19]. Although insulin tends to form amyloid aggregates [17,20], it is readily glycated [21]. Glycation of insulin by d-ribose prevents the formation of aggregates, and d-ribose-glycated insulin is highly cytotoxic. However, aggregated insulin is not cytotoxic [18]. W7FW14F apomyoglobin (W7FW14F ApoMb) forms aggregates, inducing cytotoxicity mediated by platelet-activating factor (PAF). In addition, W7FW14F ApoMb aggregates increase the expression levels of the PAF receptor. Of note, these aggregates also decrease the expression levels of PAF-acetylhydrolase (AH) type II (PAF–AH II), which degrades PAF [19]. On the other hand, human amylin forms amyloid fibrils, which are associated with damage to pancreatic islet β-cells and development of T2D [22].

## 2. Physiological Role of Amylin

### 2.1. Role of Amylin in the Central Nervous System (CNS)

Amylin inhibits food intake and delays gastric emptying, leading to the reduction of blood glucose levels and body weight. Moreover, pramlintide, an amylin analogue, also reduces body weight in humans [23]. Therefore, it is considered that amylin as well as insulin play important roles in controlling blood glucose levels. Peripherally administrated amylin reduces food intake [24,25], and amylin passes through blood–brain barrier (BBB) [26,27] to reach its binding sites, which are widely distributed in the central nervous system (CNS) [28,29]. Thus, amylin from pancreatic islet β-cells, can affect the CNS to regulate food intake and slow gastric emptying, thereby leading to a reduction in body weight.

The area postrema (AP), located in the caudal hindbrain, is considered as one of the major sites of amylin action, and ablation of the AP leads to a reduction of the inhibitory effect of amylin on food intake [30]. In addition, infusion of the selective amylin receptor antagonist AC 187 into the AP blocks reduced food intake elicited by the peripherally administrated amylin [31]. The ventral tegmental area (VTA) in the midbrain is also affected by amylin to regulate food intake, and amylin administration in the VTA reduces food intake and body weight in rats [32]. Moreover, AC 187 administration in the VTA increases food intake [33]. Peripherally administrated amylin can enhance the effect of leptin in the ventromedial hypothalamus (VMH) and arcuate nucleus (ARC) of the hypothalamus on the reduction of food intake and body weight [34,35].

Amylin functions to slow gastric emptying in rodents [36,37,38]. Furthermore, pramlintide slows gastric emptying in humans, including patients with diabetes [39,40,41,42]. Slowing gastric emptying leads to reduced nutrient delivery from the stomach to the small intestine, resulting in the slowed entry of glucose into blood. Thus, amylin can control glycemia by reducing postprandial blood glucose levels. Amylin can cause the delay of gastric emptying via the CNS [37]; it is believed that this effect may be due to stimulation of the AP, but its precise mechanism is still unclear [43]. Additionally, amylin and its mRNA [44,45] and amylin-binding sites [46] have been found in the stomach. Thus, amylin produced in the stomach and/or pancreatic islet β-cells can act on the stomach and cause the suppression of gastric emptying without CNS stimulation.

A recent study reported that in the VMH, amylin enhances leptin signaling via the induction of interleukin-6 (IL-6) and the expression of the mRNA of Lepr-b [47], which is the signaling form of the leptin receptor [48]. Leptin receptors are expressed in the VMH [49], and leptin in the VMH functions to reduce body weight [50]. Amylin increases the production of IL-6 in microglias but not in neurons or astrocytes, and IL-6 antibody administrated in the lateral ventricles diminished amylin-elicited reduction of body weight. The expression of phosphated STAT3 (pSTAT), which is induced by leptin signaling [51], is enhanced by amylin in neurons of the VMH. In addition, IL-6 antibodies and gene knockout in mice inhibit this amylin-mediated enhancement of leptin-induced pSTAT expression. Thus, amylin may enhance leptin signaling, leading to a decrease in body weight via the induction of IL-6 release from microglias.

### 2.2. Role of Amylin in Pancreatic Islet β-Cells

Amylin exerts effects on pancreatic islet β-cells as well as the CNS. Knockout of amylin results in an increase in glucose-induced insulin secretion from pancreatic islet β-cells [52] while physiological concentrations of amylin (up to 100 pM) inhibit this secretion [53]. In addition, amylin controls the proliferation of pancreatic islet β-cells depending on the glucose level; at low glucose concentrations, amylin induces the proliferation of pancreatic islet β-cells, whereas at high concentrations, it reduces the proliferation [53].

## 3. Cytotoxicity of Amylin

**Figure 3:**
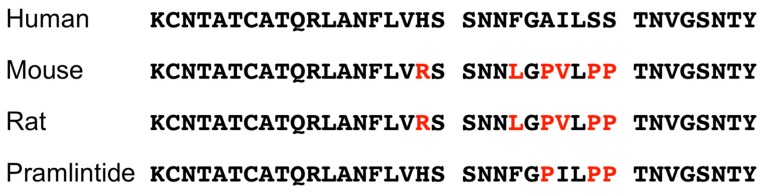
Sequence alignment of the amino acid sequences of human, mouse, and rat amylins and pramlintide. Amino acids that differ from human amylin are highlighted in red.

Amyloid aggregates composed of amylin are found in pancreatic islet β-cells of patients with T2D [54,55]. The formation of amyloid fibrils of human amylin is believed to damage pancreatic islet β-cells and be related to the development of T2D [22]. Interestingly, human amylin forms amyloid fibrils, whereas rodent amylin does not. Residues 20–29 of human amylin are responsible for the formation of amyloid fibrils [56,57], and there is a 6-amino acid difference between human and rodent amylin in this region (Figure 3). Three prolines of these six amino acid residues in rodent amylin are considered to be important differences for the prevention of the formation of β-sheet structure and amyloid fibrils. Proline substitution at positions 25 (Ala), 28 (Ser) and 29 (Ser) of human amylin decreases the stability of its β-sheet structure [58], and pramlintide, which is clinically used to treat patients with type 1 diabetes and T2D, is human amylin containing these substitutions [59] (Figure 3). Although the amino acid residues of the two β-sheet structures of human amylin differ among models, the formation of oligomers and fibrils by these β-sheet structures is common [60,61,62,63]. Fibril formation by human amylin involves three phases - a lag phase (monomers and oligomers exist), an elongation phase (elongation of fibrils), and a saturation phase (amyloid fibrils are in equilibrium) [64]. Human amylin in the lag phase are toxic to pancreatic islet β-cells as well as pancreatic islets. However, human amylin fibrils in the elongation and saturation phases exhibit decreased cytotoxicity to INS-1β-cells [64,65].

Secretory granules are acidic (approximately pH 5.5) [66]. The uncharged state of His18 in human amylin in the neutral environment leads to the aggregation of human amylin. In contrast, the charged state of His18 in human amylin in the acidic environment does not form aggregates [67,68]. Moreover, replacement of His18 in human amylin by a positively charged arginine reduces the cytotoxicity to pancreatic β-cell line MIN6 [67,68] and pancreatic islets [69]. Human amylin contains multiple basic residues and lacks acidic residues. Thus, human amylin is positively charged at neutral pH. This facilitates the interaction between human amylin and negatively charged lipids in the membrane. Moreover, the formation of human amylin fibrils is strongly promoted by negatively charged lipid bilayers [70,71,72,73]. The N-terminus of human amylin is inserted into the membrane, forming an α-helical structure [74,75]. Human amylin bound to the membrane promotes amyloid nucleation on the surface of the membrane, leading to the formation of oligomeric structures and amyloid fibrils [75,76,77,78]. Furthermore, the membrane of pancreatic islet β-cells possesses lipids carrying a negatively charged headgroup and human amylin aggregates with the membrane of INS-1β-cells [79]. In addition, non-fibrillar amylin weakly interacts with membranes containing lipids carrying phosphatidylethanolamine (PE). However, fibrillar amylin strongly interacts with these membranes and promotes their disruption, which is associated with the growth of amylin fibrils on the membrane surface [70,80]. The mutation of serine-to-glycine at position 20 (S20G) of the human amylin gene has been linked to T2D [81]. In addition, S20G amylin tends to form fibrils, induces apoptosis, and reduces the number of β-cells in pancreatic islets [82,83].

Human amylin induces cell membrane permeabilization and perturbation, endoplasmic reticulum (ER) stress, mitochondrial damage, and β-cell death [84,85,86,87,88]. Furthermore, human amylin extracellularly added to INS-1β-cells at toxic concentrations crosses the plasma membrane and approximately 50% of amylin is localized in mitochondria and 5% or less is localized in the ER [89]. Human amylin forms oligomers that subsequently form fibrils followed by amyloid deposits [90]. Human pro-amylin can also form amyloid fibrils [91] and be integrated into intracellular amyloid fibrils [92]. A processing defect of human pro-amylin is related to amyloid formation and β-cell death [93].

Human amylin oligomers are considered to be more toxic than fibrils and amyloid deposits, and oligomers may intracellularly form in pancreatic islet β-cells [22,85,94]. Human amylin oligomers in transgenic mice expressing human amylin are mostly found in insulin secretory vesicles of pancreatic islet β-cells and may be released from these impaired vesicles. Human amylin oligomers can also be detected in the cytosol, ER membrane, swollen ER and damaged mitochondria. Human amylin oligomers in the pancreatic islet β-cells of patients with T2D are more abundant than those in non-diabetic individuals [85]. Reportedly, insulin in β-cell granules prevents human amylin aggregation [95,96,97]. Human amylin oligomers can permeate the plasma membrane [98]. Furthermore, a recent study reported that amylin aggregates administered by intraperitoneal (i.p.) injection to human amylin-expressing transgenic mice induce human amylin aggregation by seeding endogenous human amylin in pancreatic islet β-cells and increase blood glucose concentration, implying that T2D may be transmissible via amylin aggregates that act like prions [99].

## 4. Protection of Pancreatic Islet β-Cells from Cytotoxicity of Amylin

**Figure 4:**
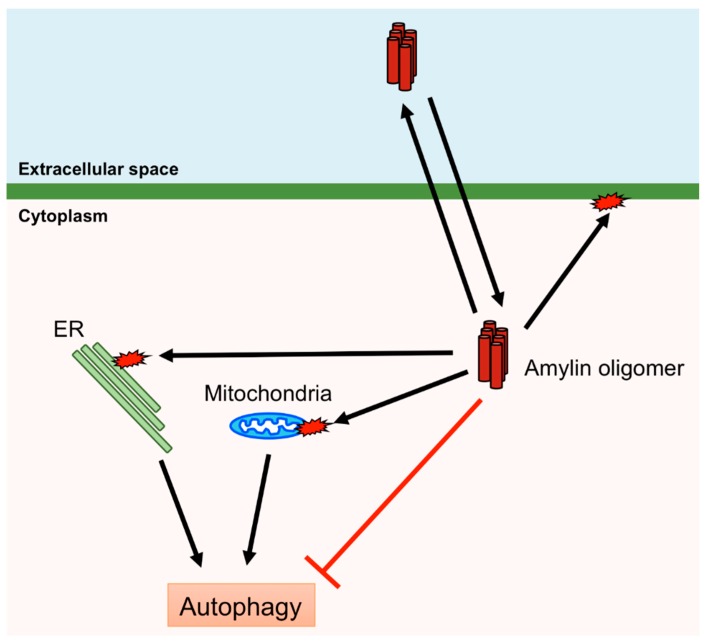
Cytotoxic effects of human amylin oligomers. Human amylin oligomers permeate and disrupt the plasma membrane and induce damage to mitochondria and the endoplasmic reticulum (ER). Autophagy can remove damaged mitochondria and the ER. However, human amylin oligomers inhibit autophagy.

Human amylin aggregates in pancreatic islet β-cells induce ER stress, mitochondrial damage, and membrane disruption, leading to β-cell death and diabetes [85,86,100]. Therefore, removal of aggregates and damaged organelles is crucial for protecting β-cells. Macroautophagy, hereafter referred to as autophagy, is bulk degradation of removing cytosolic components, including aggregated proteins and damaged organelles, and is induced by various stimuli, including the presence of aggregated proteins and organelle damage [101]. Thus, autophagy plays an important role in protecting pancreatic islet β-cells from the cytotoxicity of amylin. Autophagy is controlled by autophagy-related (ATG) proteins that transport the cytosolic components to lysosomes via autophagosome formation. Accumulation of denatured or aggregated proteins and damaged organelles activates the Unc-51-like kinase 1/2 (ULK1/2) complex or inactivates the mammalian (or mechanistic) target of rapamycin complex 1 (mTORC1), which inhibits the ULK1/2 complex. ATG16L is activated by the ULK1/2 complex and ATG12 by ATG7, and these subsequently form the ATG12–ATG5–ATG16L complex. The ATG12–ATG5–ATG16L complex conjugates PE to LC3 to form an autophagosome [102,103]. Autophagy deficiency in β-cell-specific Atg7-deficient mice expressing human amylin leads to the accumulation of oligomers and amyloids of human amylin in pancreatic islet β-cells [104]. Activation of autophagy by rapamycin, which inactivates mTORC1 that negatively regulates autophagy, inhibits β-cell death induced via overexpression of human amylin [105]. Furthermore, defects in autophagy result in β-cell death and diabetes [106]. Mitochondria change their shape via fusion and fission, and damaged mitochondria are removed via autophagy (mitophagy) [107]. A recent study demonstrated that overexpression of human amylin in INS-1 β-cells increases the fission of mitochondria, activates mTORC1 and inhibits mitophagy [108], suggesting that the accumulation of amylin leads to the inhibition of autophagy although amylin aggregates are removed by autophagy. Therefore, it may prove difficult to remove amylin aggregates once accumulated to a certain level in the β-cell (Figure 4).

Reportedly, amylin aggregates could be disaggregated by small molecules, such as β-sheet breaker peptides, chemical chaperones and foldamers [109]. β-sheet breaker peptides are synthetic inhibitors of amyloid aggregation. These peptides bind to oligomers or amyloid aggregates of human amylin and prevent the formation of amyloid aggregates. Various β-sheet breaker peptides have been reported to disaggregate amylin aggregates [110,111,112,113,114]. Proline substitution at position 26 in human amylin (I26P) destabilizes the β-sheet structure and does not form fibrils. The formation of human amylin fibrils is inhibited in the presence of I26P [110,115]. α-Aminoisobutyric acid (Aib) contains two methyl residues and has been shown to break β-sheets. Aib-NF-Aib-VH is a β-sheet breaker peptide generated through the substitution of alanine and leucine in the peptide corresponding to residues 13–18 (ANFLVH) of human amylin using Aib. In the presence of Aib-NF-Aib-VH, the formation of human amylin fibrils is blocked [111]. α,β-dehydrophenylalanine (ΔF) breaks β-sheets [116]. Similar to human amylin, a peptide corresponding to residues 22–27 (NFGAIL) of human amylin forms β-sheets and fibrils [113,117]. FGAΔFL is a pentapeptide produced by incorporating ΔF at the position of I in FGAIL and does not form β-sheets. FGAΔFL binds to human amylin and inhibits the formation of fibrils of human amylin [112]. NFGAX2L is a hexapeptide produced by incorporating 2-aminobenzoic acid (X2) at the position of I in NFGAIL, and NFX2AX2L is produced by incorporating X2 at the positions of G and I. NFGAX3L is produced by incorporating 3-aminobenzoic acid (X3) at the position of I, and NFX3AX3L is produced by incorporating X3 at the positions of G and I. All these substituted hexapeptides do not form β-sheets and inhibit the formation of human amylin fibrils [114]. 4-Phenylbutyrate (PBA), a chemical chaperone, disaggregates human amylin amyloid. In addition, PBA prevents β-cell death and recovers β-cell function [118]. Orcein-related small molecule, O4, is another chemical chaperone that induces the disaggregation of human amylin [119]. ADM-116, a pentaquinoline foldamer, can permeate the plasma membrane and prevent mitochondrial damage induced by amylin [89]. Furthermore, ADM-116 prevents the formation of amylin aggregates, thereby protecting pancreatic islet β-cells from the cytotoxic effects of amylin [120].

## 5. Conclusions

Amylin is co-stored and co-secreted with insulin and functions to control blood glucose levels by inhibiting food intake and slowing gastric emptying. However, human amylin aggregates in pancreatic islet β-cells and aggregates of human amylin induce cell death, leading to T2D. Human amylin oligomers are considered to be the most toxic form of amylin aggregates. These can cross the plasma membrane and appear to act similarly to prions. Therefore, there is the possibility that T2D could be a transmissible disease via transfer of amylin oligomers between individuals. Thus, the inhibition and disaggregation of amylin aggregates might be a viable therapeutic strategy against T2D. Further investigation of the mechanisms of amylin aggregate formation and their cytotoxic modes of action is crucial.
